# Development of Fournier’s gangrene after chemotherapy for the recurrence of testicular cancer despite the absence of anorectal lesions: A case report

**DOI:** 10.1097/MD.0000000000038688

**Published:** 2024-07-26

**Authors:** Kenichi Nonaka, Kota Kawase, Kimiaki Takagi, Yuta Takatsu, Koji Maniwa, Chika Takao, Minoru Komura, Yoshinori Mushika, Noriyuki Takeuchi, Toshio Kato, Mitsuhiko Kusakabe, Mitsutaka Kondo

**Affiliations:** a Department of Digestive Surgery, Daiyukai General Hospital, Ichinomiya, Aichi, Japan; b Department of Urology, Daiyukai Daiichi Hospital, Ichinomiya, Aichi, Japan; c Department of Surgery, Daiyukai General Hospital, Ichinomiya, Aichi, Japan; d Department of Pathology, Daiyukai General Hospital, Ichinomiya, Aichi, Japan.

**Keywords:** anorectal, case report, chemotherapy, Fournier’s gangrene, myelosuppression

## Abstract

**Background:**

Fournier’s gangrene usually occurs when a specific bacterium intrudes into soft tissue, causing a wound or tumor. We encountered a patient with Fournier’s gangrene due to severe myelosuppression after chemotherapy, despite the absence of an initial lesion on the anus and rectum.

**Case presentation:**

A 54-year-old man with a left testicular cancer recurrence had undergone chemotherapy. He had asymptomatic hepatitis and high hepatitis B virus DNA levels, which were normalized by administering tenofovir alafenamide fumarate. Twelve days after the start of chemotherapy, he complained of severe pain around the anus. The following day, he went into septic shock. Visual inspection showed dark purple skin discoloration on the left side of the anus. Laboratory data revealed severe neutropenia. Computed tomography showed a high density of soft tissue on the left side of the anus and gas bubbles in the left femoral ring. We diagnosed the patient with Fournier’s gangrene due to a severe immunosuppressive state resulting from chemotherapy. We emergently removed necrotic tissue to the fullest extent possible. However, because the patient was in severe sepsis status, careful management in the intensive care unit was required for 32 days. After the first emergency operation, we performed several additional excisions. Finally, 391 days after the initial surgery, the patient was discharged from our hospital. The tumor has not recurred, and he is under outpatient observation in the urology department.

**Conclusion:**

Fournier’s gangrene should be considered in patients who are in a severe myelosuppressive state due to chemotherapy, have normal hepatitis B virus DNA levels but high hepatitis B surface antigen after tenofovir administration, complain of severe pain in the perianal area, and have a dark purple skin discoloration around the anus, despite having no initial anorectal lesions.

## 1. Introduction

Fournier’s gangrene is a necrotizing soft tissue infection that develops mainly in the anorectal or genitourinary region and spreads rapidly to the surrounding genital, perianal, and perineal areas.^[1]^ Fournier first described it in 1883.^[2]^ Fournier’s gangrene occurs when bacteria, including streptococci, staphylococci, clostridia, coliforms, corynebacteria, and *Bacteroides*,^[3]^ invade soft tissue in a mono- or polymicrobial fashion through an entry point such as a wound (including surgical wounds) or tumor, resulting in soft tissue necrosis and septicemia. With regard to the anorectal region, this infection occurs when hemorrhoidal fistulae are present.^[4]^ Treatment may involve Thiersch operation for rectal prolapse,^[5]^ hemorrhoid treatment,^[6–9]^ or chemotherapy or chemoradiotherapy for patients with rectal cancer.^[10,11]^ Fournier’s gangrene is frequently accompanied by an underlying systemic disease, diabetes mellitus, and heavy alcohol consumption^[12,13]^; other causes include leukemia^[14,15]^ and the human immunodeficiency virus.^[16,17]^ All these conditions can be considered immune-compromising.

Necrosis is a life-threatening complication of soft tissue infection, with high morbidity and mortality (multiple organ failure). The mortality rate for Fournier’s gangrene is estimated at 16%.^[3]^ Therefore, prompt diagnosis, necrotic tissue removal, and careful systemic management are required when encountering such patients. We encountered a case of Fournier’s gangrene due to severe myelosuppression after chemotherapy, despite the absence of an anorectal lesion. We report the case here with some literature discussion.

## 2. Case presentation

A 54-year-old man underwent a left high-level radical orchiectomy for left testicular cancer 2 years earlier. The cancer’s histological type was seminoma (pT1N0M0), and its clinical stage was I. It recurred in the left retroperitoneal cavity lymph node, and the urologist planned BEP (bleomycin, etoposide, and cisplatin) therapy. However, the patient was an asymptomatic hepatitis B carrier, and 5 months before chemotherapy, the hepatitis B virus DNA (HBV-DNA) was 6.7 log/mL (the normal range is < 1.3 log/mL). Therefore, the urologist administered tenofovir alafenamide fumarate. (At our hospital, the diagnosis and treatment of viral hepatitis are handled by the gastroenterology department; they examined the patient and then instructed the urology department to prescribe the drug. The urology department prescribed this drug because we do not have a chemotherapy department, and each department administers chemotherapy and drugs to control the progression of fulminant hepatitis B.) The HBV-DNA decreased to < 1.3 log/mL 2 months before chemotherapy initiation.

After normalizing the HBV-DNA, the patient was administered 170 mg of etoposide and 34 mg of cisplatin on days 1 to 5, and 30 mg of bleomycin on days 2 and 6. Twelve days after starting chemotherapy, he complained of severe pain around the anus. On the evening of the 13th day, he went into septic shock. On the 14th day, the urology department consulted our department (digestive surgery).

**Figure 1.**
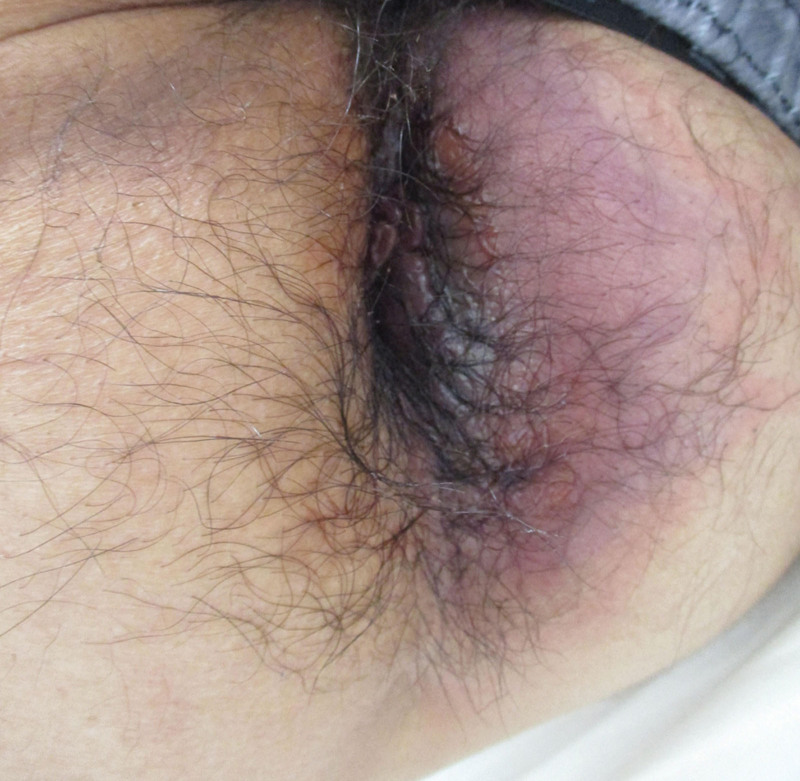
The gross perianal findings on the day of diagnosis. The skin on the left side of the anus was discolored dark purple.

**Figure 2.**
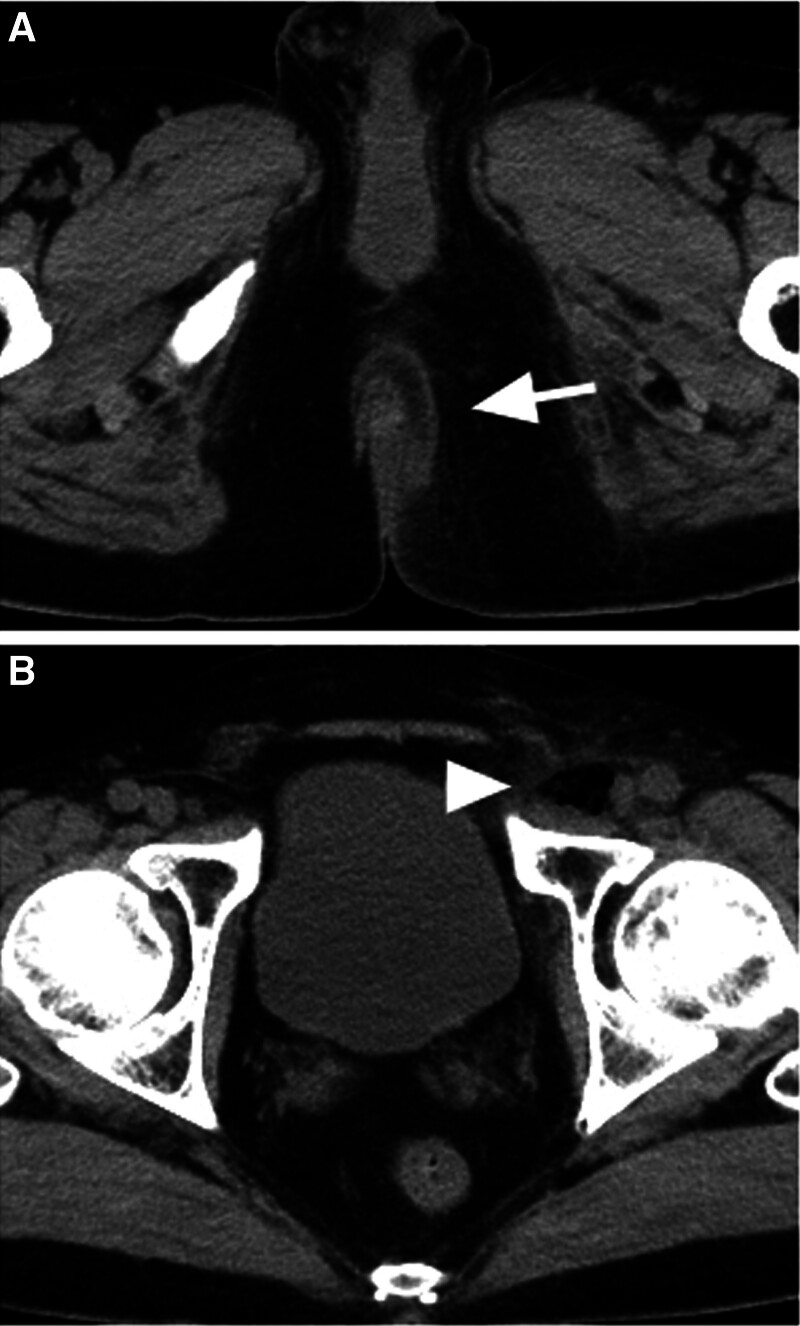
The computed tomography findings on the day of diagnosis. (A) The density of the soft tissue on the left side of the anus was elevated (white arrow). (B) Gas bubbles were found in the left femoral ring (white arrowhead).

Physical examination revealed that the left side of the patient’s anus had a dark purple discoloration (Fig. 1). A rectal examination using the index finger was performed, with no indication of hemorrhoids or neoplastic lesions. However, the patient complained of severe pain during the examination. Next, a rectal examination using a proctoscope was performed. The rectal mucosa from the upper border of the anal canal to approximately 4 cm deep was dark purple in the 3 to 7 o’clock direction. Laboratory data revealed severe leukopenia (90/μL; Laboratory Risk Indicator for Necrotizing Fasciitis [LRINEC] score = 2), thrombocytopenia (1 × 10^4^/μL; thrombocytopenia is not counted in the LRINEC score), and elevated C-reactive protein levels (31.01 mg/dL; LRINEC score = 4). Other data included hemoglobin (10.4 g/dL; LRINEC score = 2), sodium (135 mEq/L; LRINEC score = 0), and creatinine (109.6 μmol/L; LRINEC score = 0). The LRINEC score was calculated as ≥ 8 (since blood glucose levels were not measured), strongly suggesting necrotizing fasciitis.^[18]^ Computed tomography (CT) revealed an escalation of the fatty tissue density under the skin on the left side of the anus and gas bubbles in the left femoral ring (medial region of the femoral vein) (Fig. 2).

Based on these findings, we diagnosed the patient with Fournier’s gangrene due to severe myelosuppression after chemotherapy. The causative organism was thought to have invaded through the anus or the rectum and reached the left femoral ring through the left obturator foramen because gas bubbles were observed in the left femoral ring.

**Figure 3.**
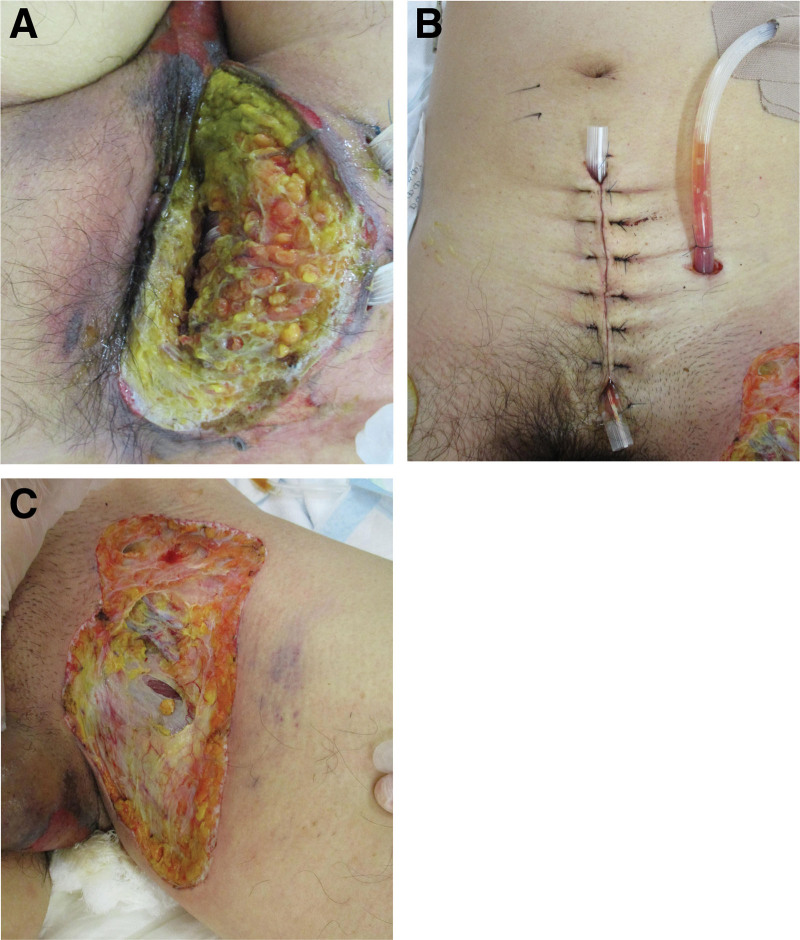
The gross findings of the operative wounds on postoperative day 4. (A) The necrotic skin and the subcutaneous tissue on the left side of the anus were removed. (B) Laparotomy, removal of the left obturator foramen, and cavity drainage were performed. (C) The skin on the medial side of the left thigh was removed due to necrosis occurring during the operation.

An emergency surgery was performed. We removed discolored skin and necrotic fatty tissue (slightly brownish compared with normal fatty tissue), and minor bleeding from under the skin was observed after the removal. Subsequently, the abdomen was opened for the removal of the left femoral ring’s necrotic tissue and the left obturator foramen’s fatty tissue, where the causative organism may have passed to the left femoral ring. The skin on the medial side of the left thigh, which was salmon pink preoperatively, was discolored dark purple, as was the skin on the left side of the anus. Therefore, the discolored skin and fatty tissue were excised (Fig. 3). A necrotic specimen obtained during surgery was submitted for bacterial culture, and *Escherichia coli* was detected. Postoperatively, the patient was managed with a ventilator and catecholamine administration in the intensive care unit (ICU).

**Figure 4.**
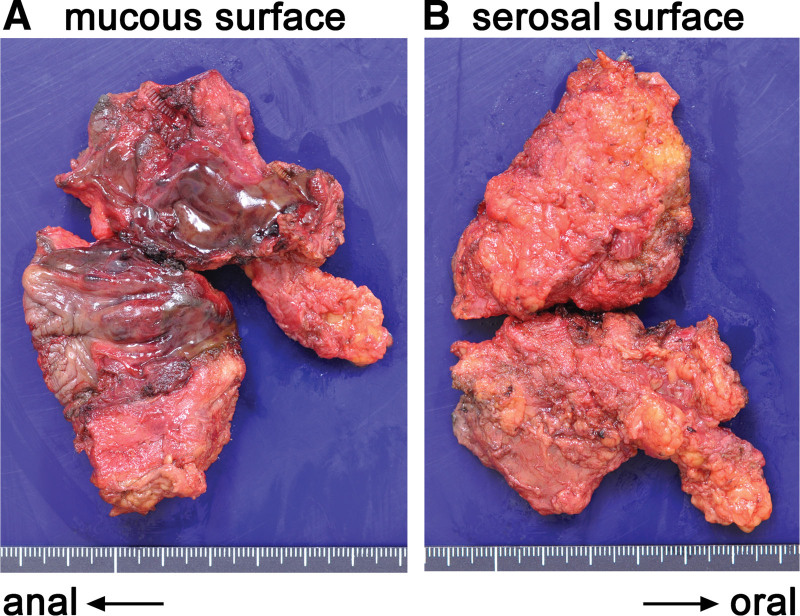
Gross findings of the resected specimen on the day of the surgery. No apparent tumor, hemorrhoidal fistula, or other soft tissue entry point was observed.

Dialysis was initiated the day after surgery due to multiple organ failures caused by sepsis. The neutropenia improved post-operation; however, additional resection of necrotic tissue and transverse loop colostomy were performed on day 4 because of continued acute circulatory failure, elevated lactic acid level, and stool adhering to the wound periphery. As the anus was almost necrotic, it was resected, and the remnant rectum was sutured closed. No tumors, hemorrhoids, or anal fistulae were observed in the resected specimen (Fig. 4).

**Figure 5.**
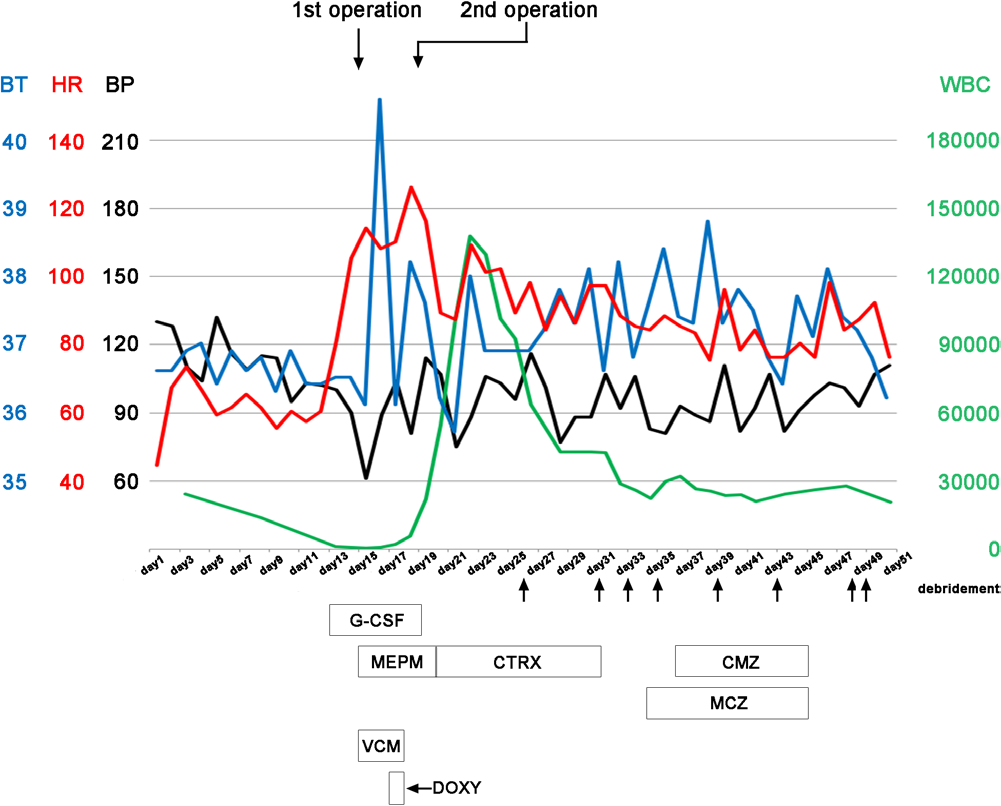
Timeline from the first day of chemotherapy to the intensive care unit discharge. After chemotherapy, significant myelosuppression was observed; on day 14, tachycardia and hypotension were noted. The pulse and blood pressure stabilized after a second surgery on day 19. BT=body temperature, BP=blood pressure, CMZ=cefmetazole, CTRX=ceftriaxone, DOXY=doxycycline, G-CSF=granulocyte-colony stimulating factor, HR=heart rate, MCZ=miconazole, MEPM=meropenem, VCM=vancomycin.

Following this second surgery, the patient’s general condition and respiratory status gradually improved, and new necrotic tissue was resected in the hospital room upon detection (debridement was performed 6 times in the ICU). The patient was weaned from the ventilator 23 days after the initial surgery, discharged from the ICU 32 days later (Fig. 5), and completed dialysis after 49 days. After the wound had settled to some extent, the dermatology department performed a skin graft. The sutured and closed rectum was eventually opened, exuding a small amount of mucus; however, the surrounding skin had no mucus adhesions. We explained to the patient that if the mucosa was severely irritated, the solution was to remove the anal side of the intestinal stoma, although the operation involved some risks. The patient chose not to have the surgery because he was not particularly troubled by his current situation and the risks involved. We decided to follow up with the patient in the present condition. Finally, 391 days after the initial surgery, the patient was discharged from our hospital.

After the operation for Fournier’s gangrene, we performed CT several times to assess the abdomen and pelvis postoperatively. The recurrent lymph node was 15 × 13 mm before chemotherapy, 12 × 11 mm 12 days after chemotherapy initiation (the day we diagnosed the patient with Fournier’s gangrene), and 7 × 6 mm 72 days after the first operation. Subsequently, the lymph node returned to its normal size, approximately 7 × 4 mm. We also checked the alpha-fetoprotein, human chorionic gonadotropin, and lactate dehydrogenase levels as tumor markers. Those markers were in the normal range before chemotherapy and did not change. For 2 years after discharge from our hospital, the lymph node size and tumor markers were assessed every 4 months in the urology outpatient clinic and every 6 months thereafter. To date, the tumor has not recurred.

## 3. Discussion

This case’s histological type was seminoma (pT1N0M0), and the clinical stage was I. The 5-year survival rate for patients with clinical stage I testicular cancer is 99%.^[19]^ The following 3 alternative therapeutic strategies exist for patients with clinical stage I seminoma after a radical orchiectomy: observation, adjuvant radiation therapy, and adjuvant chemotherapy.^[19–22]^ The recurrence rate for observation is 13–20%^[19–24]^; 6.6% of cases will recur > 6 years after the initial operation,^[21]^ and recurrence is possible even > 10 years later. However, if the disease relapses, it can be cured almost completely by starting chemotherapy at that point.

The recurrence rate for adjuvant radiation therapy is < 5%,^[25]^ although this treatment is associated with several adverse events, including secondary cancer^[26]^ and infertility.^[27]^ The recurrence rate for adjuvant chemotherapy is similar to that for adjuvant radiation therapy.^[25]^ However, it is unclear whether complications in long-term follow-up, such as secondary cancer or cardiovascular disorders, will increase. Consequently, each method has its advantages and disadvantages. Nonetheless, as the final disease-specific or overall survival rates do not differ, European guidelines consider observation the first choice.^[19,28]^

BEP therapy is the standard first-line chemotherapy for advanced testicular tumors.^[29]^ Three courses of BEP therapy are recommended if the tumor is in the good prognosis group, following the International Germ Cell Consensus Classification.^[30–32]^ In contrast, for patients with intermediate or poor prognoses, 4 courses of BEP therapy are recommended as standard treatment.^[29]^ Our patient was classified in the good prognosis group because he had a seminoma, no evidence of metastasis to other organs, and normal alpha-fetoprotein during the primary tumor’s resection.^[30]^ However, Fournier’s gangrene occurred during this patient’s first course of BEP therapy, which was subsequently discontinued. Nevertheless, as there has been no recurrence, the patient remains in follow-up.

In this case, the leukopenia after chemotherapy was so significant that necrotic areas spread rapidly. Fournier’s gangrene is a deadly disease with a reported mortality rate of 16%,^[3]^ which should be treated immediately after onset. Therefore, early diagnosis is vital, and the LRINEC score has been established for this purpose. The LRINEC score uses blood and biochemical test data to predict whether a patient has necrotizing fasciitis. A score ≥ 6 indicates a suspicion of necrotizing fasciitis, whereas a score ≥ 8 indicates a strong suspicion of necrotizing fasciitis.^[18]^ This case’s LRINEC score was ≥ 8, which was strongly indicative of Fournier’s gangrene.

If a patient is diagnosed with Fournier’s gangrene, the Fournier’s gangrene severity index (FGSI)^[1]^ and Uldag FGSI^[33]^ are tools for estimating mortality based on body temperature, heart rate, other systemic conditions, and blood test data. In this case, the scores were 11 and 17, respectively, indicating a mortality rate of 75% for FGSI ≥ 10 points and 94% for Uldag FGSI ≥ 10 points. The mortality rate of Fournier’s gangrene is also affected by the time from onset to treatment. The mortality rate for cases where surgery is performed within 12 hours of onset is 10%, compared with 50% for those treated more than 12 hours after onset.^[34]^ In this case, surgery was performed > 50 hours after the onset of significant anal pain. Consequently, this case was considered to be life-threatening.

For Fournier’s gangrene, infection source control is the primary objective, along with broad-spectrum antimicrobial therapy, aggressive and repeated surgical debridement, and intensive management.^[35]^ The guidelines published by the Infectious Diseases Society of America state that empiric antibiotic therapy should be broad-spectrum (vancomycin or linezolid plus piperacillin/tazobactam or carbapenem or ceftriaxone and metronidazole) because the etiology is polymicrobial (mixed aerobic and anaerobic microorganisms) or monomicrobial (group A streptococci, community-acquired methicillin-resistant *Staphylococcus aureus*).^[36]^ In addition, the spectrum of antimicrobials should be customized based on the blood or tissue culture test results.^[36]^ For this patient, we administered meropenem and vancomycin, subsequently changing from vancomycin to cubicin because of impending renal failure. We de-escalated to ceftriaxone when *E coli* was detected in the tissue culture.

Regarding surgical debridement for a patient with Fournier’s gangrene, the necrotic tissue should be removed as soon as possible.^[37]^ If sufficient time has not elapsed since the tissue necrosed, determining the extent of resection is difficult because of the lack of color change. In such cases, necrotic tissue should be excised until the bleeding site is exposed.^[38]^ The wound should be carefully monitored postoperatively, and additional resections should be performed if necrotic areas remain or emerge. After the necrotic areas are entirely removed, skin grafting,^[39,40]^ negative pressure wound therapy (also known as vacuum-assisted closure therapy),^[41,42]^ and hyperbaric oxygen therapy can be performed to epithelialize the wound. Negative pressure wound therapy can remove tissue exudate, reduce local edema, enhance neovascularization, and improve the tissue’s natural self-healing.^[43,44]^ Conversely, hyperbaric oxygen therapy improves tissue perfusion, promotes angiogenesis and fibroblast proliferation, and increases collagen synthesis and oxygen levels in tissues.^[45]^

Four major questions can be raised in this case. The first question is whether the patient had Fournier’s gangrene due to myelosuppression after chemotherapy, as we have argued, or whether Fournier’s gangrene developed first, followed by sepsis and then leukocyte depletion. However, we could not identify a gateway for bacterial entry, such as a tumor or wound in the anorectal region. Furthermore, if sepsis occurred after Fournier’s gangrene, in this case, the bacteria would have to reach the soft tissues before strong myelosuppression appeared; however, it would be impossible for the bacteria to pass through the immune barrier of the intestinal tract without tumors or other lesions. Moreover, if sepsis occurred first, fever and tachycardia would have been present before the onset of grade 3 myelosuppression (12 days after the first day of chemotherapy). Therefore, in this case, leukopenia does not appear to have resulted from sepsis.

Conversely, post-chemotherapy myelosuppression generally begins 7 to 10 days after the initiation of chemotherapy and is at its lowest 10 to 14 days later. In this case, grade 3 leukopenia occurred 12 days after the onset of chemotherapy, and grade 4 leukopenia occurred 14 days after chemotherapy. After the emergence of severe myelosuppression, the patient had shock symptoms such as tachycardia and hypotension. Therefore, we concluded that chemotherapy-induced leukopenia caused Fournier’s gangrene.

The second question is how the bacteria reached the soft tissues. Cisplatin has been shown to rarely cause gastrointestinal ulceration or perforation of the gastrointestinal tract as an adverse event, which may be a gateway for bacterial invasion. However, in this case, no obvious rectal ulcers or perforations were observed in the resection specimen. In addition, the CT scan showed gas in the left medial femoral ring, although it was far away from the rectum. No gas or abscess was noted between the gases in the left medial femoral ring and the rectum. Therefore, we hypothesized that bacteria entered from the lower rectum; however, because the rectal wall is covered with rectal proper fascia and the surrounding area contains parietal pelvis fascia, adipose tissue, and lymphatic tissue, if a perforation of the anorectum had occurred, abscesses and gas would be expected to first accumulate around the rectum. Gas would present from the rectum to the left femoral ring in a connected or sporadic manner, especially as > 50 hours had passed between disease onset and surgery. Thus, the most likely situation is that the gas or abscess would accumulate around the rectum and progress to the medial side of the femoral ring. However, during surgery, we removed as much tissue as possible between the rectum and the gas reservoir inside the left femoral ring, where the bacteria could have passed, and no abscess was observed, as would occur with gastrointestinal tract perforation. Therefore, the possibility that Fournier’s gangrene was caused by perforation of the gastrointestinal tract, an adverse event caused by cisplatin, can almost be ruled out.

Perianal abscess is another disease that presents with perianal pain and shows a hyper-absorbable area around the anus on CT, which should be differentiated. Perianal abscess is a disease where the anal glands become infected with bacteria and form abscesses. However, with this condition, the skin tone is unchanged or accompanied by redness, and fluid pus accumulates in the soft tissue. In this case, the severe leukopenia caused by chemotherapy may have allowed bacteria to invade through the anal glands, which would usually be prevented. However, this case differs from typical cases of Fournier’s gangrene because no initial injury or lesion was found, especially in the anorectal region.

The third question is whether myelosuppression caused by chemotherapy was the only cause of Fournier’s gangrene. This patient was an asymptomatic carrier of hepatitis B. When chemotherapeutic or immunosuppressive drugs are administered to such patients, nucleic acid analogs such as tenofovir should be administered prophylactically to prevent the reactivation of HBV and the development of fulminant hepatitis. In this case, the amount of HBV-DNA before chemotherapy reached the target level by quantitative testing, and indeed, fulminant hepatitis did not occur after chemotherapy. However, the hepatitis B surface (HBs) antigen was 1581.56 IU/mL (the normal range is < 0.05 IU/mL). The HBV has been shown to suppress neutrophil extracellular trap (NET) release by modulating reactive oxygen species production and autophagy.^[46]^ These NETs are extracellular fibrous structures comprising chromatin, histones, neutrophil elastase, myeloperoxidase, cathepsin G, proteinase 3, and other complement proteins that bind and kill various microbes by exposing them to high concentrations of NET-related microbicidal factors, thereby preventing microbial spread. Therefore, the normal level of HBV-DNA with the high HBs antigen level did not necessarily indicate low levels of HBV itself, which may have suppressed the release of NETs from neutrophils and weakened their resistance to the bacteria.

The fourth question is whether this case is pseudo-gas gangrene or not. CT images of pseudo-gas gangrene show gas in the soft tissues, resembling necrotizing fasciitis. However, the gas is not generated by gas-producing bacteria. The gas is caused by the rectovaginal fistula^[47]^ or gastrointestinal perforation (due to irradiation after the resection of rectal cancer^[48]^ and ulcer,^[49]^ among others), which allows gas in the gastrointestinal tract to enter the soft tissue through the perforation or by trauma,^[50,51]^ which can allow air from outside the body to penetrate the soft tissue. When this patient visited our department for consultation, we conducted a rectal examination and proctoscopy and found no obvious rectal perforation or ulcer. Additionally, the patient had no history of irradiation of the abdomen. Therefore, it is unlikely that gas in the gastrointestinal tract entered the soft tissues. However, when this case was diagnosed as Fournier’s gangrene and emergency surgery was performed, the skin color of the left inguinal region changed to dark purple, and necrosis had begun to appear, suggesting that gas-producing bacteria had reached this region. Based on the above information, this case is considered to be a case of Fournier’s gangrene rather than pseudo-gas gangrene.

A PubMed search using the keywords “cancer chemotherapy” and “Fournier’s gangrene” yielded 23 cases. There were 16 cases of cancer chemotherapy purely associated with the development of Fournier’s gangrene.^[10,11,52–62]^ Among them, bacteria invaded the soft tissue from the rectum in 10 cases.^[10,11,52–56,60,61]^ Nine cases had rectal cancer,^[10,11,52–55,60,61]^ and the remaining 1 developed Fournier’s gangrene after bevacizumab was used to treat resected colorectal cancer.^[56]^ Bevacizumab was also used in 4 of the 9 rectal cancer-bearing cases mentioned above. Bevacizumab may cause the formation of microthrombi in the arteries, which may be the cause of Fournier’s gangrene. Bevacizumab is frequently used as chemotherapy for rectal cancer; however, it is necessary to be extremely cautious about the development of Fournier’s gangrene. Indeed, the above 10 cases were different from the report of marked leukopenia after chemotherapy, causing Fournier’s gangrene. Of the remaining 6 cases, 1 had Fournier’s gangrene of the scrotum and penis during chemotherapy for gastric cancer.^[62]^ The other 1 developed perforation of the small intestine after chemotherapy, including bevacizumab, for local recurrence in the pelvis and metastasis in the periaortic lymph nodes after rectal cancer surgery.^[57]^ Therefore, the above 2 cases are different from ours. The remaining 4 cases were all patients with hematologic malignancies.^[58,59]^ These patients underwent chemotherapy and experienced severe myelosuppression, which was followed by Fournier’s gangrene. These 4 cases are relatively similar to the present case, except that the portal of entry for the bacteria was the genitourinary tract in 3 cases^[58,59]^ and a laceration wound outside the anus in 1 case.^[58]^ The urogenital organs were thought to be the entry point for the bacteria in these 3 cases because in 2 cases, ulcers appeared on the scrotum before Fournier’s gangrene developed,^[58]^ and in the other case, edema appeared on the scrotum.^[59]^ The organisms responsible for the infection were also different: *Pseudomonas aeruginosa* in the above 4 cases and *E. coli* in the present case. Based on the above information, there have been no reports of cases similar to ours.

The anorectal region, which is constantly exposed to bacteria, is a strong barrier; therefore, it is almost impossible for bacteria to penetrate without a lesion. Indeed, we found no report of a case similar to the one presented here. In this case, the immune barrier of the rectal mucosa or anal canal epithelium likely became dysfunctional due to severe leukopenia caused by chemotherapy and the HBV-mediated suppression of NET release. Thus, the bacteria invaded the soft tissues and caused Fournier’s gangrene, despite the absence of any lesions in the rectum or anus.

This study had some limitations. First, the absence of glucose measurements for Fournier’s gangrene diagnosis. This was because we did not know glucose was included in the LRINEC score when ordering the blood test. In the future, all test items included in the LRINEC score should be ordered to make a prompt diagnosis. Second, we found no lesions that could serve as a gateway for bacterial entry. Invasion through the anal glands remains a possibility, although if this were the case, a perianal abscess should have been present. Regardless, the leukopenia caused by chemotherapy would have allowed the bacteria to invade through the anal glands, making this case extremely rare.

### 3.1. Patient perspective

The patient understood and was comfortable with the treatment he received. After a colostomy was created, the patient had a mucous fistula in the area where the anus used to be; however, the amount of mucus coming out of the fistula was small and did not significantly interfere with the patient’s daily life.

## 4. Conclusion

The diagnosis of Fournier’s gangrene should be considered when a patient with severe leukopenia from chemotherapy and normal HBV-DNA levels but high HBs antigen after tenofovir administration complains of severe pain in the perianal region and dark purple discoloration of the perianal skin, despite no initial perianal lesion.

## Acknowledgments

The authors would like to thank Editage for the English language review.

## Author contributions

**Conceptualization:** Kenichi Nonaka.

**Methodology:** Kenichi Nonaka.

**Project administration:** Kenichi Nonaka.

**Software:** Kenichi Nonaka.

**Supervision:** Kenichi Nonaka, Mitsuhiko Kusakabe, Mitsutaka Kondo.

**Validation:** Kenichi Nonaka, Mitsuhiko Kusakabe, Mitsutaka Kondo.

**Visualization:** Kenichi Nonaka.

**Writing – original draft:** Kenichi Nonaka, Yuta Takatsu.

**Writing – review & editing:** Kenichi Nonaka, Kota Kawase, Kimiaki Takagi, Koji Maniwa, Chika Takao, Minoru Komura, Yoshinori Mushika, Noriyuki Takeuchi, Toshio Kato, Mitsuhiko Kusakabe, Mitsutaka Kondo.
